# Effects of Humans on Behaviour of Wildlife Exceed Those of Natural Predators in a Landscape of Fear

**DOI:** 10.1371/journal.pone.0050611

**Published:** 2012-11-28

**Authors:** Simone Ciuti, Joseph M. Northrup, Tyler B. Muhly, Silvia Simi, Marco Musiani, Justin A. Pitt, Mark S. Boyce

**Affiliations:** 1 Department of Biological Sciences, University of Alberta, Edmonton, Alberta, Canada; 2 Department of Fish, Wildlife, and Conservation Biology, Colorado State University, Fort Collins, Colorado, United States of America; 3 Alberta Innovates, Technology Futures, Vegreville, Alberta, Canada; 4 Faculty of Environmental Design, University of Calgary, Calgary, Alberta, Canada; Federal University of Parana (UFPR) ) – Campus Palotina, Brazil

## Abstract

**Background:**

Human disturbance can influence wildlife behaviour, which can have implications for wildlife populations. For example, wildlife may be more vigilant near human disturbance, resulting in decreased forage intake and reduced reproductive success. We measured the effects of human activities compared to predator and other environmental factors on the behaviour of elk (*Cervus elaphus* Linnaeus 1758) in a human-dominated landscape in Alberta, Canada.

**Methodology/Principal Findings:**

We collected year-round behavioural data of elk across a range of human disturbances. We estimated linear mixed models of elk behaviour and found that human factors (land-use type, traffic and distance from roads) and elk herd size accounted for more than 80% of variability in elk vigilance. Elk decreased their feeding time when closer to roads, and road traffic volumes of at least 1 vehicle every 2 hours induced elk to switch into a more vigilant behavioural mode with a subsequent loss in feeding time. Other environmental factors, thought crucial in shaping vigilance behaviour in elk (natural predators, reproductive status of females), were not important. The highest levels of vigilance were recorded on public lands where hunting and motorized recreational activities were cumulative compared to the national park during summer, which had the lowest levels of vigilance.

**Conclusions/Significance:**

In a human-dominated landscape, effects of human disturbance on elk behaviour exceed those of habitat and natural predators. Humans trigger increased vigilance and decreased foraging in elk. However, it is not just the number of people but also the type of human activity that influences elk behaviour (e.g. hiking vs. hunting). Quantifying the actual fitness costs of human disturbance remains a challenge in field studies but should be a primary focus for future researches. Some species are much more likely to be disturbed by humans than by non-human predators: for these species, quantifying human disturbance may be the highest priority for conservation.

## Introduction

Understanding the effects of human disturbance is critical for effective management and conservation of wildlife in an increasingly human-dominated world. The human population has exploded in recent decades with a subsequent acceleration in demands for resources [1]. This demand has led to a growing and increasingly pervasive network of roads that extends the reach of humans into wildlife habitats [2], [3]. Human activity along road networks can have an impact on wildlife behaviour that is often complex and varies among species and across both space and time [4]–[6]. Indeed, it is not just the number of people but the type of human activity that is expected to cause shifts in behavioural responses of wildlife: for instance, previous studies suggested that certain hunting modalities and motorized recreational activities can have a stronger impact on wildlife than less intrusive disturbances [6]–[10]. However, the actual effect of human disturbance on behaviour, population dynamics and life history are still poorly documented [9], [11]–[13].

With the exception of large protected areas, where it is relatively easy to measure and control human disturbance, human-dominated landscapes are complex environments where it can be challenging to disentangle all sources of human disturbance. Within large protected areas, anti-predator behaviour of wildlife is often primarily shaped by natural predators [14], [15], but little is known about shifts in anti-predator behaviours when humans are likely to be the major driving force. Humans can have a different influence on wildlife depending on land-use management – for instance whether hunting or motorized recreational activities are permitted [7], [16]. Measurements of the types and levels of human disturbance and their effects on wildlife are needed not only to guarantee functional ecosystems in currently human-dominated landscapes [3], [17], but also to plan conservation policies for those remote areas where human exploitation of resources is expected [1].

Behaviour can indicate how animals respond to disturbance in their environment [11], [18]. For instance, vigilance, or scanning of the environment, is adopted by a wide range of birds and mammals to increase the probability of detecting predators or other sources of disturbance – including humans [18]. Thus, vigilance responses can be a useful way to measure disturbance of wildlife. Vigilance patterns are indeed finely shaped by predator presence [19], human disturbance [20], habitat characteristics [21], and prey group size and composition [22], including age differences among individuals of the group [23]. Vigilance levels may also increase in males during the mating season [15] and in females with offspring [24]. The principal cost of vigilance is thought to be time, where opportunities for alternative behaviours are forfeited [18] with the most common trade-off between vigilance and foraging [25]. Most models assume that vigilance is incompatible with foraging and that, across species, time spent vigilant is usually inversely correlated with time spent feeding [26]. In regard to ungulates, it is still unclear how much feeding time is actually lost due to vigilance because ungulates are capable of maintaining their rate of food intake despite being vigilant, because of their ability to scan the environment while chewing vegetation [27], [28]. Although foraging costs of vigilance are likely less important than traditionally assumed [26], vigilance certainly induces some foraging costs [29], [30]. Empirical studies suggest that disturbance and related vigilance in ungulates can reduce reproductive success and potentially impact populations [31]–[35].

Here we disentangle the effects of human disturbance (i.e., vehicle traffic) [3], [36], [37] and different types of human activities on the vigilance behaviour of elk (*Cervus elaphus* Linnaeus 1758) in southwest Alberta, Canada. Alberta has a growing road network due to increased demands for resources and recreation, which creates concern for wildlife conservation (*e.g.*, for grizzly bears *Ursus arctos* Linnaeus 1758 see [38]). Roads pose a major risk to many animal populations [39]–[40], and elk are a good model species because they are of keen economic and social interest across North America and Europe and may act as a charismatic flagship species [41] in conservation and management actions. A few studies have shown the effect of human disturbance on the behaviour of elk [7], [42], [43], while several have focused on the effect of natural predators on the behaviour of this species [24], [44], [45]. However, the majority of these studies were performed during short periods (e.g. summer, rut) or within single-use human activity areas (e.g., protected areas), given that it is challenging to collect behavioural data where hunting is permitted and ungulates adopt more secretive behaviour [6]. Thus, here we analysed original elk behavioural data collected across a range of land-use types and seasons in the same population subjected to a variety of management policies. We documented for the first time the effect of fine-scale traffic patterns on the behaviour of a large herbivore across an entire road network.

## Methods

### Ethics Statement

Our data collection complied with all relevant federal laws of Canada and provincial laws of Alberta. Procedures adopted in this study were reviewed and approved by the University of Alberta Animal Care and Use Committee ACUC – Biosciences (Animal care protocol # 536-1003 AR University of Alberta, Edmonton, Canada), by all jurisdictions of the Alberta Government (Permit Numbers: BI-2008-19, RC-06SW-001 and 23181CN), and by Parks Canada (Permit Numbers: WL-2010-7292, WL-2010-5755).

### Study Area

The study occurred within a montane ecosystem along the eastern slopes of the Rocky Mountains in southwest Alberta, Canada. This is a diverse landscape, ranging from flat agricultural grasslands, in the east, to mixed conifer/hardwood forests and mountains, in the west. The study area (∼5000 km^2^) was composed of private agricultural land, public land (a.k.a. Crown land of Canada), and a national park (Waterton Lakes National Park). Private land in the eastern half of the study area was dominated by cattle ranches where recreational use and other activity was controlled and restricted by landowners. Activities in the public land were uncontrolled and dominated by recreational use including all-terrain vehicle (ATV) use, hunting, fishing, and hiking, with only a small fraction of road traffic related to natural gas extraction. Activities in the national park were strictly controlled and limited along designated paths.

### Elk Behavioural Observations

From June 2010 to May 2011, observations were carried out at dawn and dusk using binoculars (10×50) and spotting scopes (25–40×60) to observe elk within open areas of the national park, private and public land. Observations were performed by the same 2 observers (SC, SS) from roads without leaving the vehicle at a distance always greater than 500 meters (distance between the observers and elk herd, mean ± SE: 729.8±60.1 m in public land, 752.83±49.4 m in the national park, and 957.3±31.6 m in private land). No elk were observed on public land from winter to early spring, when elk were usually on private land or in the national park (winter range). We defined a herd as a group of elk with a nearest-neighbour distance of less than 100 m, regardless of their behavioural state [23]. We recorded date, time, location, herd size and sex and age class composition for each herd observed. The exact position of each herd was assessed with the combined use of a GPS (eTrex Legend, Garmin International Inc., Olathe, KS, USA), a compass and a rangefinder (elite 1600 Arc, Bushnell, Overland Park, KS, USA). We divided elk into three age–sex classes. “Mothers” were defined as adult females with a nursing calf present. “Females” were defined as adult females with no nursing calf present. “Yearlings” were markedly smaller females and those males with only one antler point per side. Male vigilance is known to be partly devoted to looking for competitors (i.e. other males) for a long period of time before, during and after the rut [15]. We thus collected data on female dominated herds only (i.e., males <50%, [23]; males were adults with two or more antler points per side). We divided elk activity into six mutually exclusive behavioural states [23] as follows: feeding - standing or walking slowly with the head below the level of the shoulder; scanning - standing with the head at or above the shoulder level; travelling - walking, trotting or running with the head at or above the shoulder level; grooming - licking or scratching oneself or another; aggression - kicking, biting or charging another with head fully raised; and resting - any behaviour while lying on the ground.

For each herd observed we recorded both group vigilance and individual vigilance. Group vigilance was estimated using a group scan sampling rule and a fixed-interval, time-point recording rule [46]. We used a voice recorder to note the behaviour (feeding, scanning, travelling, grooming, aggression, and resting) of each member of the herd from left to right at the instant of the scan sample signal. The fixed-point interval was 15s for herds of fewer than 15 individuals and was extended to 30s, 45s or 60s for larger herds. We took 30 samples (usually 7.5 min total duration) for each herd scanned. Group vigilance was estimated as the percentage of time- intervals where at least one elk was scanning. Group vigilance accounts for the number of bedded individuals. Several elk resting within open areas for long time is a clear sign of low group vigilance levels.

Individual vigilance was estimated by a focal animal sampling rule and a continuous recording rule [46]. Active focal individuals were selected from the herd based on their age–sex class (females, mothers, and yearlings, as defined above) and position in the herd (peripheral or interior). We defined interior animals as those that a predator from outside the herd could not approach without first encountering another herd member [47]. Peripheral animals were those individuals that could first be encountered by a predator that approached from outside the herd [47]. According to Childress and Lung [23], we assigned to each focal elk the distance to the nearest-neighbour elk (inter-individual distance). To reduce the probability that the same individual elk was observed more than once, only one to four individuals were observed in each herd. Each focal individual was observed for 15 min, until they were no longer visible, or they began to rest, whichever came first. Observations for less than 3 min were excluded from the analysis [23]. Behavioural states were recorded using a voice recorder. We used the free software JWatcher v.0.9 (http://www.jwatcher.ucla.edu/) to digitize and process voice records. We calculated scan frequency (number of scans/min) and the proportion of time scanning (time scanning/time active) for each individual. We also computed proportion of time travelling (time travelling/time active), proportion of time grooming (time grooming/time active), proportion of time feeding (time feeding/time active, i.e. foraging efficiency), and the average length of foraging bouts. The more an elk interrupts feeding behaviour to scan the landscape, the shorter the expected length of foraging bouts.

We recorded vigilance behaviours in elk that attempt to reduce their probability of being selected as a prey and to detect the predator (e.g. humans and natural predators) at a safe distance. We did not investigate elk behaviours that try to deter the predator when it is encountered. Observations carried out when hunters or grizzly bears were observed chasing elk groups were discarded from analyses due to low sample size.

### Vehicle Traffic and Human Land Use Data

Fifty-two randomly distributed traffic counters (Apollo, Diamond Traffic Products, Oakridge, OR, USA) were deployed on trails and a variety of road types (i.e., paved roads, gravel roads, unimproved roads, truck trails and ATV trails). Moreover, 21 trail cameras (Silent Image RM30, RECONYX, Creekside, WI, USA) were deployed at randomly selected locations on roads and trails. Trail cameras provided time-stamped photographs of motorized use that triggered the camera’s infrared sensor. Pictures of motorized vehicles were used to quantify traffic. Using this large dataset, Northrup et al. [48] modeled traffic volume for the entire road network in our study site. Traffic volumes were modelled for 3 seasons: summer, hunting, and winter-spring (see below for further details on how we defined seasons).

The location of each elk herd was assigned the following two road variables: the linear distance to the nearest road (km) and the density of roads (km/km^2^) in a 3 km-wide circular buffer. The buffer area was chosen based on average daily mobility of elk in the area computed as the sum of distances between successive GPS locations recorded for *n* = 168 collared elk monitored in this area (MS Boyce, unpublished data). We classified roads based on disturbance rate (traffic volume thresholds) following an exponential fashion:

- all roads, regardless of traffic volumes (i.e. even considering roads estimated by the traffic models to be travelled by 0 vehicles per day);
- only roads with a traffic volume of at least 1 vehicles every 8 hours (at least 3 vehicles per day),
- only roads with at least 1 vehicle every 4 hours (at least 6 vehicles per day),
- only roads with at least 1 vehicle every 2 hours (at least 12 vehicles per day),
- only roads with at least 1 vehicle every hour (at least 24 vehicles per day),
- only roads with at least 1 vehicle every 30 minutes (at least 48 vehicles per day),
- only roads with at least 1 vehicle every 15 minutes (at least 96 vehicles per day),

We calculated the distance from the closest road – or the density of roads around a 3-km wide buffer – for each elk location and traffic class based on the above volume thresholds, resulting in 14 different road variables (7 for distance from roads, 7 for density of roads).

Forty-three trail cameras (RECONYX, Creekside, Wisc.) were deployed on roads and trails at randomly selected locations [49]. We used data derived from 32 cameras located in the areas (public land *n* = 19, private land *n* = 13) where we sampled elk behavioural observations to calculate the average daily occurrence of recreational land users, *i.e.*, hikers, bikers, equestrians and ATV users.

Based on trail camera data, ∼95% of motorized traffic recorded within public lands was related to recreational activities (cars, trucks, RVs: ∼80%; ATVs such as quads and motorbikes: ∼15%), while only ∼5% was related to industrial activities (natural gas extraction). About 99% of motorized traffic recorded within the national park was of recreational type (cars, trucks, RVs), while ∼95% of motorized traffic recorded within private lands was related to recreational and ranching activities with only a small fraction of ATVs (cars, trucks, RVs: ∼93%; ATVs: ∼2%).

### Definition of Seasons According to Human Disturbance

The study area was seasonally visited by thousands of tourists and recreationists. We thus divided the study period into specific recreational periods, i.e. summer, hunting, and winter-spring (Table 1). Intense recreational use occurred during summer from late May through early September both in public lands and the national park, although with extremely different modalities – i.e., strictly managed and limited along paths in the national park, uncontrolled on public lands, where ATVs, free camping and activities both on and off trails were permitted. Activities on private lands were similar to those recorded on public lands (e.g. ATVs, camping) but restricted by landowners. Hunting seasons were mostly limited to early September through the end of November (Table 1). There were no restrictions on the number of licensed hunters having access to public land, while access to private land was strictly controlled by landowners. Hunting was not allowed in the national park, whereas hunting was allowed immediately outside its borders from early September through late February (Table 1). Although elk in Waterton Lakes National Park are not actively hunted within the park, animals are hunted along park boundaries when elk use lower-elevation areas during fall and winter due to shallow snow and higher forage availability. This is the reason why elk in this park do not show signs of habituation, as previously suggested for this specific area by St. Clair and Forrest [42]. During the winter-spring season, from December 1^st^ to late May, recreational use of this area was largely absent, irrespective of the local land management (Table 1).

**Table 1:** Seasonal and spatial variation of human disturbance recorded in a complex multi-use landscape of SW Alberta, Canada.

	Summer			Hunting			Winter-Spring*	
	(late May through early September)			(early September through the end of November)			(December through late May)	
	Public land	Private land	National Park	Public land	Private land	National Park	Private land	National Park
**HUNTING**	X	X	X	√	√^LR^	X^ PIO^	X	X^ PIO^
**ATVs**	√	√^LR^	X	√	√^LR^	X	X	X
**Human presence^1^ mean vehicles per day**±**SE**	167±11	64±3	436±32	157±10	90±5	338±25	27±1	60±4
**vehicles per hour**	7	3	18	7	4	14	1	2

[X: not allowed; **√**: allowed; **^LR^** landowner restricted; **^PIO^** permitted immediately outside park’s borders; **^1^** average number of vehicles having access to areas where elk behavioural observations were performed, as recorded by road counters; *elk were not observed on public lands in winter-spring when they usually move to lower elevations within private lands and the national park].

### Wolf and Grizzly Bear Resource Selection Functions (RSFs)

Wolf (*Canis lupus* Linnaeus 1758) and grizzly bear are predators of elk in this area. Cougars (*Puma concolor* Linnaeus 1771) are also present, though predation upon elk in this area is rare (based on *n* = 10 GPS collared cougars monitored in this area, J. E. Banfield unpublished). We used pre-existing models for the spatial distribution of wolves and grizzly bears in the study area (for wolves: [50]; for grizzly bears: Foothills Research Institute Grizzly Bear Project, S. E. Nielsen unpublished data). In both cases, satellite-telemetry location data from wolves and bears were obtained, and population-averaged resource selection functions (RSFs) were developed. RSFs are any function proportional to the probability of selection of a resource unit, and have been widely used to model species distributions [51], [52]. RSFs were estimated using logistic regression, where resource units at telemetry locations are compared to resource units at random landscape locations [53]–[55]. Wolf and bear home ranges were estimated using a 95% kernel density estimator [56] of telemetry location data. The “two-stage” method was followed to calculate population-level RSFs [57]–[58] where an RSF is calculated for each individual animal and these are averaged across all individuals. Predictive ability of the RSF models was evaluated using k-fold cross validation [59].

### Data Analyses

We modelled group vigilance and scan frequency of focal individuals using linear mixed models with Gaussian distributions of errors. The dependent variables were transformed (arcsine square root [group vigilance]; ln[scan frequency +1], where ln = natural logarithm) to improve normality of residuals and reduce skew. We included the identity code of each herd as a random intercept in our mixed models to account for replicated observations on the same herd through time [60]. Herds were identifiable given that more than 100 individuals in the area were individually recognizable by numbered and coloured ear tags and fitted with GPS radiocollars at the time of this study. Following Burnham et al. [61], we constructed 14 *a priori* mixed models based on biological relevance and field observations using 6 variables to predict group vigilance, and 17 *a priori* mixed models using 9 variables to predict scan frequency in focal individuals. Predictor variables were defined as follows:

#### Human disturbance:

1) land-use/season, i.e. a dummy variable resulting from the combination of 3 seasons (summer, hunting, and winter-spring) with 3 land management strategies (public land, private land, national park). Based on location and date, one of the following codes was assigned to each elk observation: national park-summer, national park-hunting, national park-winter-spring; private land -summer, private land-hunting, private land-winter-spring; public land-summer, public land-hunting. No elk were observed on public land during winter-spring;
2) road variables (distance or density) based on traffic volume thresholds.
Natural predators:
3) wolf RSF and grizzly bear RSF.
Elk anti-predator strategies (within-group factors):
4) ln [herd size], where ln = natural logarithm;
5) inter-individual distance;
6) within-group position, i.e. an elk peripheral or interior to the herd;
7) age-sex class, i.e. females, mothers, yearlings.
Anti-predator strategies (environmental factors):
8) distance from the nearest tree cover, with tree cover characterized by a canopy cover ≥25%;
9) terrain ruggedness [62].

All variables were used to build the set of *a priori* models predicting scan frequency in focal individuals, while specific variables related to focal elk (predictor variables 5, 6, and 7) were excluded when we modelled group vigilance. Herd size was included in all *a priori* models to control for the effect of group size on both group vigilance and scan frequency [63].

The models best predicting group vigilance and scan frequency were identified by minimum AIC, model ranking and weighting [61], [64]. The use of AIC to select the best model could be problematic when using mixed models given that AIC penalizes models according to the number of predictor variables [65], which is not clear because of the random effect. Both Gelman and Hill [66] and Bolker et al. [67] noted this and advocate for the use of the deviance information criterion (DIC) in such instances. As a consequence, we also examined our model selection using the DIC approach. A likelihood ratio based R^2^ [68] was used as an approximate measure of explained variation in the mixed models, according to the formula *R*_LR_^2^ = {1-exp[−2*n* ^−1^(*L*_M_-*L*_0_)}]/{1-exp(2*n* ^−1^ *L*_0_)} where *L*_M_ is the log-likelihood of the model of interest, *L*_0_ is the log-likelihood of the intercept-only model and *n* is the number of observations.

Each set of *a priori* models predicting group vigilance was built using only 1 of the 14 road variables described above (7 variables for the distance from nearest road, 7 variables for road density). We ran these 14 independent sets of models to identify which road variable was the best predictor of group vigilance (based on AIC, verified with DIC). After this step, we presented the set of models built with the best road variable in predicting group vigilance. We repeated the same procedure when we modeled scan frequency in focal elk.

We were aware of the potential for spatial autocorrelation in our data given that close observations were suspected to have similar vigilance levels as a response to similar environmental factors. Indeed, Diniz-Filho et al. [69] stressed that AIC is particularly sensitive to the presence of spatial autocorrelation and may generate unstable and overfit minimum adequate models to describe ecological data. We ran a Mantel test [70] which showed no spatial autocorrelation in either the dataset dealing with group vigilance (Mantel test based on 9999 replicates, *r_M_* = −0.006) or scan frequency (*r_M_* = 0.022).

We observed elk from roads without leaving the vehicle. Prior to generating our sets of *a priori* models, we examined whether our data on group vigilance were affected by the presence of observers in some way. Thus, we fit a linear mixed model to test for the effect of the distance between the observer and the elk herd (measured with a rangefinder) on group vigilance, taking into account wind strength and wind direction which might increase the probability of the observer being spotted by the elk. We included the identity code of each herd as a random intercept in our mixed models to account for replicated observations on the same herd through time [60]. Wind strength was measured with an anemometer, while wind direction was measured with a compass and computed as deviation from 0 in degrees, with 0 degrees being the direction of the wind blowing from the observer and the herd.

We used least squares linear regression to test the effect of various types of human users recorded by cameras (average daily presence of hikers, bikers, equestrians, and ATV users) on elk behavioural patterns (average scan frequency, proportion of time grooming, proportion of time scanning, and proportion of time travelling) during summer and the hunting season on private lands and public land. We also used linear regression to test the effect of scan frequency on length of foraging bouts, proportion of time feeding (i.e. foraging efficiency), and proportion of time travelling in focal elk.

All analyses were performed with R 2.14.1 [71]. All GIS analyses were performed with ARCMAP 9.2 (ESRI Inc., Redlands, CA).

## Results

We made 424 direct observations of elk herds (15,032 elk) and 870 observations of focal individuals from June 2010 to May 2011. We observed 124 groups (220 focal individuals) in summer, 92 groups (154 focal individuals) during the hunting season, and 208 groups (496 focal individuals) during winter and spring. We observed 212 mothers, 336 adult females without calves, and 322 yearlings. Calf/female ratios - including within females both adult females and mothers - were (mean ± SE) 0.22±0.07 on public lands, 0.32±0.02 in the national park, and 0.37±0.01 on private lands. Female productivity recorded on public lands was lower than that in the national park (independent samples t-test, t = −2.336, p = 0.022) and on private lands (t = −3.436, p = 0.001).

We did not find a significant effect of the distance between observer and herd or wind direction and strength on group vigilance (linear mixed model: effect of distance between observer and herd *t* = −0.252, *p* = 0.800; effect of wind strength: *t* = 0.100, *p* = 0.920; combined effect of wind strength with wind direction: *t* = −1.035, *p* = 0.301).

### Human Disturbance Exceeded other Factors in Triggering Increased Vigilance in Elk

A comparison of models predicting group vigilance is reported in Table 2 (upper panel). The best model explained approximately 83% of the variability of group vigilance. Taking into account herd size, the top-ranked model included both the predictor variables of human disturbance, i.e. land-use/season and distance from the nearest road with a traffic volume of at least 12 vehicles per day. The distance from the nearest tree cover was also included in the best model. The selection of the top-ranked model using AIC was confirmed using DIC (Table S1, upper panel). Models including the variable for the nearest road with a traffic volume of at least 12 vehicles per day consistently outperformed models excluding this term (Table S2).

**Table 2:** Sets of models predicting group vigilance and scan frequency in elk.

Model #	Dep. variable: arcsine square root [group vigilance], n = 424 elk groups	AIC	ΔAIC	w_i_	ER	logLik
**1**	**ln[herd size]+land-use/season+dist. nearest tree cover + dist. nearest road** **(≥12 vehicles per day)**	**118.6**	**0**	**0.9003**	**1**	**−45.3**
2	ln[herd size]+land-use/season+dist. nearest road (>12 vehicles per day)	124.2	5.6	0.0547	16	**−**49.1
3	ln[herd size]+land-use/season+ dist. nearest road (>12 vehicles per day)+Terrain ruggedness	125.4	6.8	0.0301	30	**−**48.7
4	ln[herd size]+land-use/season+ dist. nearest road (>12 vehicles per day)+wolf RSF+grizzlybear RSF	127.0	8.4	0.0134	70	**−**48.5
5	ln[herd size]+land-use/season+dist. nearest tree cover	131.3	12.7	0.0015	600	**−**52.7
6	ln[herd size]+land-use/season	141.7	23.2	<0.0001	10^5^	**−**58.9
7	ln[herd size]+land-use/season+ wolf RSF+grizzly bear RSF	142.3	23.7	<0.0001	10^5^	**−**57.2
8	ln[herd size]+land-use/season+ Terrain ruggedness	143.7	25.2	<0.0001	10^5^	**−**58.9
9	ln[herd size]+dist. nearest road (>12 vehicles per day)	190.7	72.2	<0.0001	10^15^	**−**89.4
10	ln[herd size]+dist. nearest tree cover	203.2	84.6	<0.0001	10^18^	**−**95.6
11	ln[herd size]	203.9	85.3	<0.0001	10^18^	**−**96.9
12	ln[herd size]+Terrain ruggedness	205.2	86.6	<0.0001	10^18^	**−**96.6
13	ln[herd size]+wolf RSF+grizzly bear RSF	206.4	87.8	<0.0001	10^19^	**−**96.2
14	Intercept only	379.5	261.0	<0.0001	10^56^	**−**185.8
**Model #**	**Dep. variable: ln[scan frequency +1], n = 870 focal elk**	**AIC**	**ΔAIC**	**w_i_**	**ER**	**logLik**
**1**	**ln[herd size]+land-use/season + dist. nearest road (**≥**12 vehicles per day)**	**75.3**	**0**	**0.9476**	**1**	**−24.7**
2	ln[herd size]+land-use/season+inter-individual distance	81.9	6.5	0.0361	26	**−**27.9
3	ln[herd size]+land-use/season+wolf RSF+grizzly bear RSF	84.5	9.1	0.0098	97	**−**28.2
4	ln[herd size]+land-use/season+ dist. nearest tree cover	87.6	12.3	0.0020	464	**−**30.8
5	ln[herd size]+land-use/season	88.1	12.8	0.0016	587	**−**32.0
6	ln[herd size]+land-use/season+age/sex class	88.6	13.3	0.0012	758	**−**30.3
7	ln[herd size]+land-use/season+within-group position	89.3	13.9	0.0009	1062	**−**31.6
8	ln[herd size]+land-use/season+Terrain Ruggedness	89.8	14.5	0.0007	1396	**−**31.9
9	ln[herd size]+dist. nearest road (≥12 vehicles per day)	173.4	98.1	<0.0001	10^21^	**−**80.7
10	ln[herd size]+wolf RSF+grizzly bear RSF	190.9	115.6	<0.0001	10^25^	**−**88.5
11	ln[herd size]+age/sex class	191.7	116.3	<0.0001	10^25^	**−**88.8
12	ln[herd size]+inter-individual distance	194.8	119.5	<0.0001	10^25^	**−**91.4
13	ln[herd size]+dist nearest tree cover	195.9	120.5	<0.0001	10^26^	**−**91.9
14	ln[herd size]	197.4	122.0	<0.0001	10^26^	**−**93.7
15	ln[herd size]+Terrain Ruggedness	199.0	123.7	<0.0001	10^26^	**−**93.5
16	ln[herd size]+within-group position	199.3	124.0	<0.0001	10^26^	**−**93.7
17	Intercept only	315.4	240.1	<0.0001	10^52^	**−**153.7

Two sets of linear mixed models fit to predict group vigilance (upper panel) and scan frequency (lower panel) in elk observed in SW Alberta, Canada. Best models (in bold, first rows) explained 83% of the variability of group vigilance and 86% of variability of scan frequency, respectively, as approximated by a likelihood ratio R_LR_^2^. [*AIC* = Akaike information criterion; *ΔAIC* = difference in AIC value between the AIC of a given model and the best model (lowest AIC); *w_i_* = Akaike weights; *ER* = evidence ratio; *logLik* = log-likelihood value].

**Figure 1:**
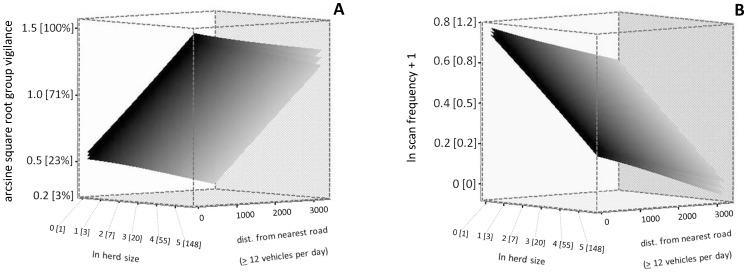
Effect of herd size and distance from nearest road on elk vigilance levels. Effect of ln herd size and distance (in meters) from the nearest road with a traffic volume of at least 12 vehicles per day on a) arcsine square root group vigilance (n = 424 groups) and b) ln (scan frequency +1) (n = 870 focal individuals) in elk observed in SW Alberta, Canada. Back transformed data are indicated within square parentheses.

According to predictions of the best model, group vigilance increased as the herd size increased (β = 0.182, SE = 0.011, Fig. 1a), confirming that larger groups have a higher probability of at least 1 elk scanning every 15 seconds. Large units (50 or more elk) were characterized by a higher probability of at least 1 elk scanning every 15 seconds (n = 96, mean ± SE, 85.7±1.7%), and had more than twice the probability of such behaviour than smaller units (3 or less elk; n = 74, 36.3±2.7%). Group vigilance increased when the distance from the nearest road decreased (i.e. when elk were closer to roads; β = −0.132, SE = 0.034, Fig. 1a). Indeed, group vigilance increased by 23% in magnitude from groups that were observed greater than 1000 m from a road (54.8±3.2%, n = 80) compared to groups observed less than 250 m from roads with a traffic volume of at least 12 vehicles per day (67.7±2.7%, n = 108). Group vigilance also increased as the distance from the nearest tree cover increased (β = 0.157, SE = 0.057). Group vigilance was 58.5±2.7% (n = 129) for groups less than 10 m from the closest tree line, while it was 70.8±4.4% (n = 40) for those greater than 500 m from tree line.

Coefficients and standard errors estimated by the best model for the land-use/season variable are reported in Table 3 (upper panel). The highest level of group vigilance was recorded on public land during the hunting season. Group vigilance recorded on public land in summer was higher than that recorded in the national park during winter-spring and hunting season, and on the private lands during winter-spring. Intermediate values of group vigilance were recorded on private lands during summer and hunting season. The lowest value of group vigilance was recorded in the national park during summer (Table 3, upper panel). Group vigilance increased by 84% in magnitude from groups observed in the national park in summer (47.9±4.9%) to those observed on public land during the hunting season (88.3±4.3%).

**Table 3:** Effect of spatial and temporal variation of human disturbance on elk vigilance levels.

	Land-use/season variable	β	SE
High group vigilance	Public land – hunting	0	
	Public land – summer	**−**0.37	0.12
	PRIVATE LAND – hunting	**−**0.51	0.12
	PRIVATE LAND – summer	**−**0.55	0.12
	*National Park* – winter-spring	**−**0.72	0.15
	*National Park* – hunting	**−**0.72	0.15
	PRIVATE LAND – winter-spring	**−**0.76	0.12
Low group vigilance	*National Park* – summer	**−**0.92	0.15
	**Land-use/season variable**	**β**	**SE**
High scan frequency	Public land – hunting	0	
	Public land – summer	**−**0.20	0.08
	PRIVATE LAND – summer	**−**0.25	0.08
	PRIVATE LAND – hunting	**−**0.36	0.09
	*National Park* – hunting	**−**0.38	0.10
	*National Park* – winter-spring	**−**0.47	0.10
	*National Park* – summer	**−**0.50	0.10
Low scan frequency	PRIVATE LAND – winter-spring	**−**0.51	0.08

Coefficients and standard errors (β±SE) estimated for the land-use/season variable by the best linear mixed effect models (see Table 2) predicting group vigilance in 424 elk groups (upper panel) and scan frequency (lower panel) in 870 focal elk observed in SW Alberta, Canada. The land-use/season dummy variable was derived from the combination of 3 seasons (summer, hunting, and winter-spring) with 3 different management strategies (public land, private land, and national park). No elk were observed in the Public land during winter-spring. All coefficients are in reference to the public land during the hunting season.

A comparison of models predicting scan frequency of focal elk is reported in Table 2 (lower panel). The best model explained 86% of the variability of scan frequency and, taking into account herd size, included only those predictor variables related to human disturbance, i.e. land-use/season and distance from the nearest road with a traffic volume of at least 12 vehicles per day. The selection of the top-ranked model based on AIC was confirmed by the DIC analysis (Table S1, lower panel). Models including a variable for the distance from the nearest road with a traffic volume of at least 12 vehicles per day consistently outperformed models excluding this term (Table S3).

According to predictions of the best model, the scan frequency in focal individuals increased when the herd size decreased (β = −0.067, SE = 0.008, Fig. 1b) or when the distance from the nearest road (≥12 vehicles per day) decreased (β = −0.095, SE = 0.024, Fig. 1b). Scan frequency was (mean ± SE) 0.48±0.04 scan/min (n = 264) when distance from the nearest road with at least 12 vehicles per day was ≥1000 m, while scan frequency was 0.52±0.03 scan/min (n = 264) when the distance from the nearest road was 500 to 1000 m, 0.60±0.03 scan/min (n = 230) when the distance was 250 to 500 m, and 0.72±0.04 scan/min (n = 185) when the distance was 0 to 250 m.

Coefficients and standard errors estimated by the best model for the land-use/season variable are reported in Table 3 (lower panel). The highest scan frequency level in focal individuals was recorded on public land during hunting season (mean ± SE: 1.27±0.02 scan/min). This was higher than that recorded both on public (1.16±0.07 scan/min) or private land (1.02±0.06 scan/min) during summer (Table 3 lower panel). Lowest scan frequencies for focal individuals were recorded in the national park during winter-spring (0.44±0.03 scan/min) and summer (0.51±0.04 scan/min), and on private land in winter-spring (0.38±0.02 scan/min). Intermediate values of scan frequency were recorded during the hunting season in both the national park (0.65±0.07 scan/min) and on private land (0.68±0.05 scan/min) (Table 3, lower panel).

### ATVs Exceeded Any other Human Land-use Type in Triggering Increased Vigilance in Elk

We did not find a significant effect of the number of bikers or equestrians recorded by motion-activated cameras (n = 32) on the 4 behavioural variables recorded in focal individuals (Table 4). An increase in the number of hikers was responsible for a significant increase of the proportion of time travelling in focal individuals (*R^2^* = 0.55, *p* = 0.002), but it did not affect other behavioural states (Table 4). An increase of the number of ATV users was responsible for a significant increase of scan frequency (*R^2^* = 0.35, *p* = 0.004) and of proportion of time scanning (*R^2^* = 0.52, *p*<0.001) by focal individuals, while it resulted in a significant decrease in proportion of time grooming (*R^2^* = 0.32, *p* = 0.007). The number of ATV users did not affect the proportion of time travelling by focal individuals (Table 4).

**Table 4:** Effect of different human use types on behaviour of elk.

	scan frequency	grooming	scanning	travelling
**HIKERS**	**−**0.009±0.034 *^ns^*	**−**0.005±0.008 *^ns^*	**−**0.025±0.035 *^ns^*	**0.109**±**0.027** ^**^
**BIKERS**	**−**0.983±1.317 *^ns^*	0.117±0.337 *^ns^*	0.672±1.435 *^ns^*	**−**0.779±1.161 *^ns^*
**EQUESTRIANS**	0.055±0.190 *^ns^*	**−**0.012±0.048 *^ns^*	0.145±0.201 *^ns^*	**−**0.126±0.228 *^ns^*
**ATVs**	**0.067±0.020** ^**^	**−0.017±0.005** ^**^	**0.078±0.017** ^***^	0.011±0.025 *^ns^*

Effect of different human use types – number of hikers, bikers, equestrians, and All Terrain Vehicles (ATV) users spotted by 32 motion activated cameras (public land n = 19, private land n = 13) – on 4 behavioural patterns recorded for focal elk (ln [scan frequency +1]; arcsine square root proportion of time grooming, scanning and travelling) observed during summer and hunting season in SW Alberta, Canada. The effect (β+SE) of each relationship was reported as estimated by linear regression [*ns*: not significant *(p>*0.4 in all cases); *: 0.05<*p*<0.01; **: 0.01<*p*<0.001; ***: *p*<0.001].

### Decreased Foraging Time by Elk from Increased Vigilance

**Figure 2:**
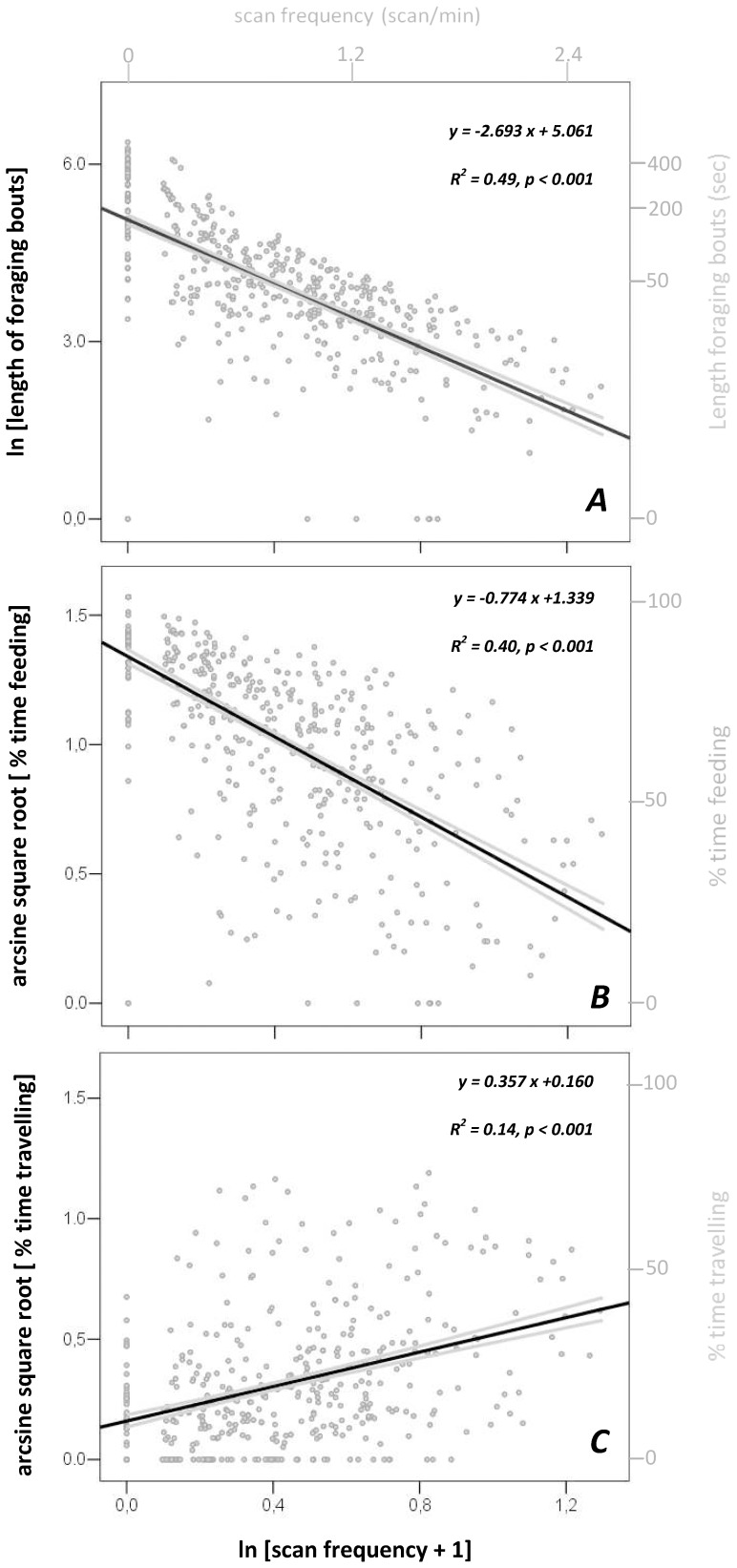
Effect of scan frequency on proportion of time feeding and travelling in elk. Effect of scan frequency (bottom x-axis, ln[scan frequency +1]; see top x-axis for back transformed data) on a) ln [length of foraging bouts], b) arcsine square root [proportion of time feeding], and c) arcsine square root [proportion of time travelling] in 870 focal elk observed in SW Alberta, Canada. Right y-axes represent back transformed data. Black lines in each graph represent linear relationships, while grey lines represent 95% confidence intervals of mean. Linear regression equations, R^2^ values and *p*-values are reported for each graph.

Variation of scan frequency recorded among 870 focal elk explained 49% of the variability in the length of foraging bouts (linear regression analysis, Fig. 2a), 40% of the variability in total feeding time (i.e., foraging efficiency Fig. 2b), and 14% of the variability in total travelling time (Fig.2c). Increased scan frequencies were significantly related to a decrease in the length of foraging bouts, a decrease in total feeding time, and an increase in total travelling time by focal individuals (Fig. 2).

**Figure 3:**
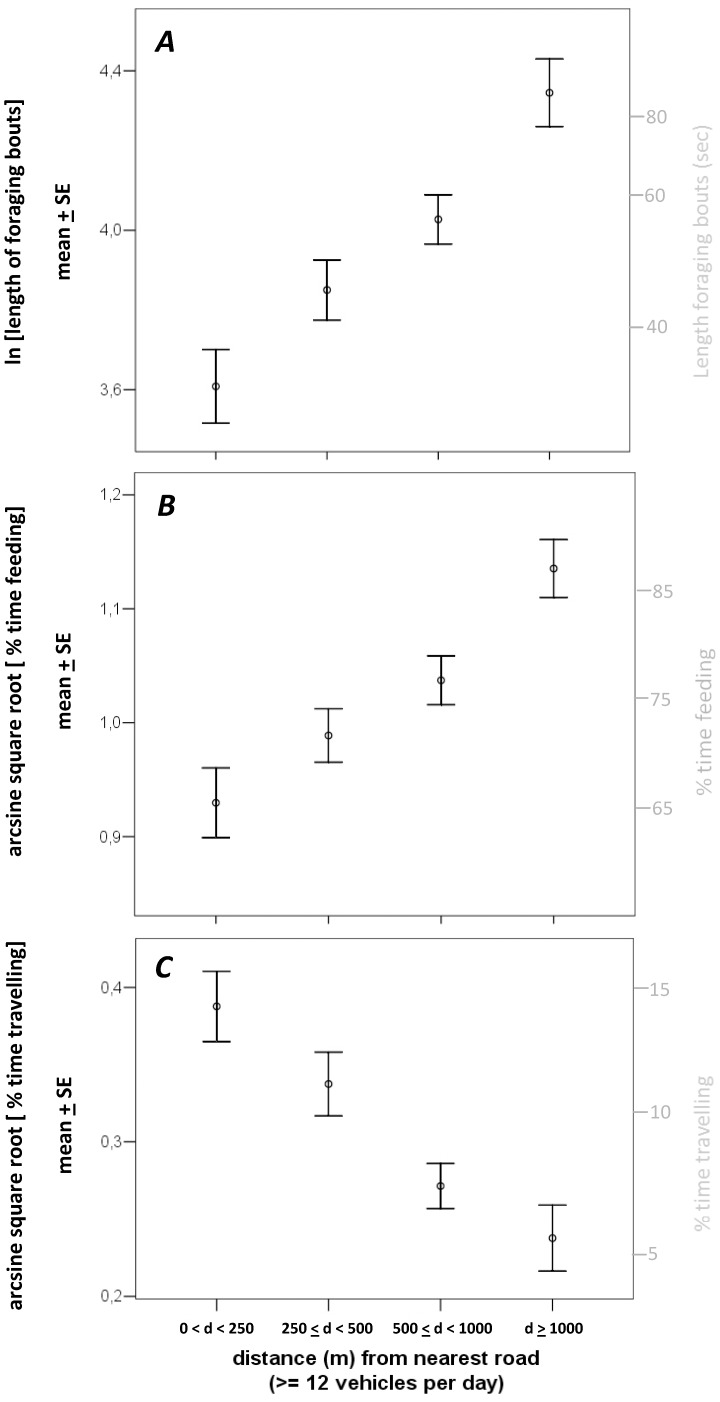
Effect of distance from nearest road on behaviour of elk. Effect of the distance from the nearest road with a traffic volume of at least 12 vehicles per day on a) ln [length of foraging bouts], b) arcsine square root [proportion of time feeding], and c) arcsine square root [proportion of time travelling] in 870 focal elk observed in SW Alberta, Canada. Right y-axes represent back transformed data. The sample size was distributed as follows: n = 188 elk (0< *d* <250 meters, where *d* is the distance from the nearest road with a traffic volume of at least 12 vehicles per day), n = 230 elk (250≤ *d* <500 meters), n = 264 elk (500≤ *d* <1000 meters), and n = 188 elk (*d* ≥1000 meters).

Scan frequency strongly influenced the other behavioural states (Fig. 2). In turn, scan frequency was shaped by the distance to the nearest road with traffic volume of at least 12 vehicles per day (Table 2 lower panel, Fig. 1b). All behavioural states were clearly shaped by the distance from roads as well. We show the magnitude of such effect in Fig. 3. Length of foraging bouts were at least 2-times longer in elk observed at distances greater than 1 km from roads than those observed close to roads (<250 m, Fig. 3a). The same applied to feeding time (Fig. 3b), with an increase of at least +20% of total feeding time by elk observed greater than 1 km from roads. Elk observed closer to roads (<250 m) increased at least +10% the time spent travelling than those elk observed greater than 1 km from roads (Fig. 3c).

## Discussion

### Effects of Humans on Elk Behaviour Exceed those of Natural Predators and of other Environmental Factors

In a human-dominated landscape, we found that the effects of humans in shaping behaviour of elk exceed those of habitat and natural predators. Factors commonly thought to be primary drivers of vigilance behaviour in elk – terrain ruggedness, natural predators, position in the group, inter-individual distance, reproductive status of females [15], [23], [47] – may play a lesser role within human-dominated landscapes. In our analysis the only habitat variable that affected vigilance levels was the distance from tree cover. This strong habitat effect was evident for groups but not individuals, meaning that elk increased scan duration (i.e., higher likelihood of at least one elk scanning in the group) but not scan frequency (i.e. number scans/min) as the distance from safe habitat increased.

We documented elk behaviour across different land-use types (treatment areas) and seasons in the same population subjected to a variety of management policies. To date, no studies have collected behavioural data within protected areas, private lands and public lands simultaneously. This is a true landscape of fear, where each human is perceived by elk to be a potential predator, even within the protected area, as animals are threatened by hunting pressure immediately along its borders. We measured actual human use on roads in these treatment areas, and we documented the effect of fine-scale traffic patterns on the behaviour of a large herbivore across an entire road network. Road traffic volumes of at least 1 vehicle every 2 hours (12 vehicles per day) induced elk to switch into a more alert behavioural mode (increased vigilance) with an actual loses in feeding time. We expected a higher traffic volume threshold although we could not make precise predictions based on previous research because this is the first study in which vigilance data have been associated with estimates of traffic volumes. In this landscape of fear, where humans are perceived by elk to be potential predators, extremely low traffic volumes were sufficient to trigger a behavioural response by elk.

We found the highest levels of elk vigilance on public lands during the hunting season, when hunting and intrusive recreational activities occurred cumulatively, whereas the lowest levels were found in the national park in summer – even when crowded with people – and on private lands during winter-spring – when human activities were almost absent after hunting season. Both public lands and national park were crowded with people in summer, but our study clearly showed that it was not just the number of people but above all it was the type of human activity that shaped elk behaviour. More people can have less of an effect if the type of human activity is relatively benign, i.e., the effect of hikers on elk behaviour in the national park was definitely lower than that of motorized recreational activities occurring on public lands in summer. ATVs and intrusive summer recreational activities can have strong influence on elk behaviour as recently documented by Naylor et al. [7]. Our camera data collected outside the national park confirmed this pattern, showing that motorized vehicles had a stronger impact than non-motorized activities (hikers, bikers, equestrians) on elk behaviour. Among non-motorized activities, bikers and equestrians had no effect on elk behaviour likely because they are more predictable and rarely leave roads and trails. In contrast, hikers evoked an increase of proportion of time travelling in elk. This response is likely linked to the flight behaviour in elk, confirming that humans on foot are more evocative than other more predictable stimuli [6]. Running in elk can be an extreme alarm behaviour, and future studies should consider this behavioural category separated from walking to better disentangle the effect of different human types on elk behaviour.

Interestingly, we found levels of vigilance on private land during summer and the hunting season that were intermediate to those recorded in the public land and the national park, reflecting that access restrictions imposed by landowners lead to lower disturbance on elk compared with the uncontrolled human disturbances occurring in the public land. Vigilance levels in the national park during the hunting season, when hunting was allowed immediately along its borders, were not different than recorded on private lands when hunting was permitted there.

### Management and Conservation of Wildlife in Human-dominated Landscapes

**Figure 4:**
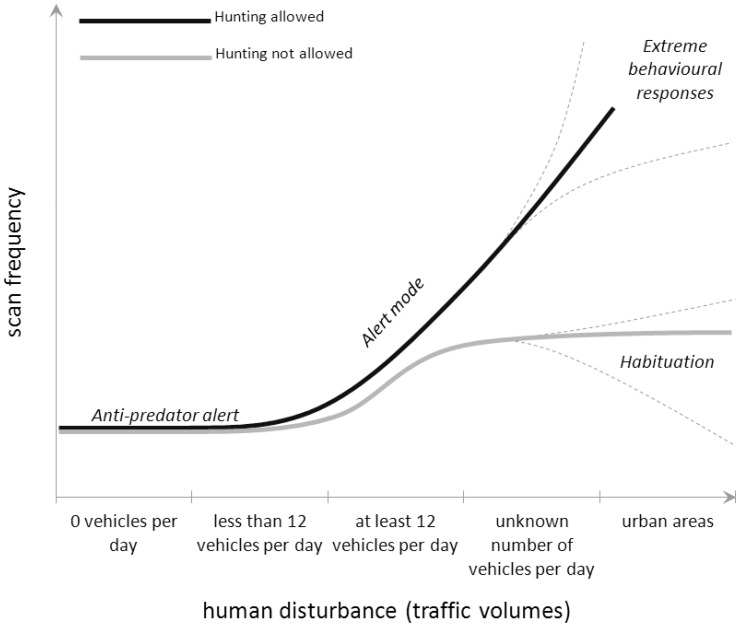
Theoretical relationship between traffic volumes and vigilance in elk. Theoretical model describing the relationship between a proxy of human disturbance (traffic volumes) and the scan frequency in elk. A constant distance (<500 m) from the nearest road and a constant habitat (open area) for each elk observed were assumed. Elk are assumed to switch to the alert mode when the nearest road has a traffic volume of at least 12 vehicles per day. Higher traffic volumes (still unknown thresholds) are predicted to have different impacts on elk behaviour depending on whether the population is hunted or not, respectively.

If we assume no human disturbance (traffic volumes of 0 vehicles per day, Fig. 4), we can expect a base level of vigilance due to natural predators, habitat characteristics and group size and composition [15], [23]. The traffic model developed by Northrup et al. [48] for our study site is the first detailed characterization of motorized human use along an entire road network to be used to evaluate large mammal behaviour. Our results showed that elk occupying areas close (e.g., <500 m) to roads (Fig. 4) switch into a more-alert behavioural mode (increased vigilance) when traffic surpasses 12 vehicles per day. This does not necessarily mean elk are displaced, but this level of traffic clearly leads to a significant rearrangement of the time spent in other activities such as feeding. High traffic volumes can have different impacts that depend on whether hunting is allowed or not (Fig. 4). If hunting is not permitted, then behavioural adaptations, such as habituation, can evoke a decrease in vigilance levels (Fig. 4; [72]). Human developments can affect the distribution of predators [73], [74]. In such cases, high human activity can even displace predators, and create spatial refuge for prey that can benefit from reduced predation risk [72], [75]. However, in a human-dominated landscape where hunting is allowed, behavioural responses to road traffic can be extreme (Fig. 4), potentially leading to high vigilance levels (elk and bison: [76]; Tibetan antelope *Pantholops hodgsonii* Abel 1826: [77]), increased flight distance (elk: [78]; other ungulates: [6]), increased movement rates (elk: [7]), and, eventually, displacement from areas surrounding roads and thus habitat loss (moose *Alces alces* Linnaeus 1758: [79]; woodland caribou *Rangifer tarandus caribou* Gmelin 1788: [80]; mule deer *Odocoileus hemionus* Rafinesque 1817: [81]; grizzly bear: [48]; elk [82], [83]).

Increased vigilance has a cost in terms of decreased feeding time, as we showed with our data, and this certainly causes reduced feed intake [18], [25], [29], [30]. We showed how elk re-arrange activity patterns depending on the distance to roads where traffic surpasses 12 vehicles per day, with more frequent interruptions of foraging bouts, decreased feeding time and increased travelling time. If disturbed, we showed that elk can even reduce certain behaviours such as grooming to minimum levels as the presence of motorized vehicles increases. Vigilance and activities such as resting or grooming are incompatible [84].

We documented the complex link between disturbance and behavioural response in a human-dominated landscape, though we were not able to estimate the actual cost of human disturbance on wildlife in terms of fitness and population dynamics. Behavioural responses by elk to the risk of predation by wolves have been shown to be correlated with increased vigilance, reduced foraging and food intake [23], [45], [47], [85]. Parallel to these behavioral responses, physiological data have revealed a decrease in the quantity of food obtained by elk in the presence of wolves and changes in the composition of their diet that exacerbate nutritional deficits in winter [86], [87]. Ultimately, predation risk induced decreased fecal progesterone concentrations [34] and decreased calf recruitment in elk [47], [87], [88]. Thus, the actual cost of predation risk by natural predator has already been documented, at least for the wolf-elk predator prey system, but not for ecological contexts where humans are a major source of disturbance for wildlife. The effects of predation risk by humans arguably could be similar to those of natural predators, given that prey have evolved anti-predator responses to threatening stimuli including lethal (e.g. hunting) and non-lethal (e.g. noises or approaching vehicles) human disturbance. Animal responses are likely to follow the same principle used by prey encountering predators [11], [89].

According to the rationale “higher disturbance – lower reproductive success” [35], we could expect lower female reproductive success in sites most heavily disturbed by humans in our study area. In fact, we recorded the lowest calf female/ratio in public lands, where we recorded higher vigilance levels than elsewhere. However, the complex link between female productivity and disturbance has yet to be fully documented. Risk effects can be manifest by reduced survival, growth, or reproduction [34], [90]–[93]. Experiments allow risk effects to be clearly identified and quantified [94], [95], but this task is more difficult in field studies [91]. The link between increased human disturbance and reduced fitness in wildlife has been only partially documented in the field for few mammal and bird species [31]–[33], [96]. The cost of disturbance is a waste of time and energy [97], and some species are much more likely to be disturbed by humans than by non-human predators ([98], [99], this study). For these species, quantifying human disturbance and its fitness cost may be the highest priority for conservation. Human factors can affect behaviour, habitat use and distribution of wildlife with potential consequences on trophic cascades [100]: these factors should play a more prominent role in conservation or wildlife management within human-dominated landscapes.

## Supporting Information

Table S1 **Sets of models predicting group vigilance and scan frequency in elk (ranked using DIC).**
(DOCX)

Table S2 **Selection of the best road variable predicting group vigilance in elk.**
(DOCX)

Table S3 **Selection of the best road variable predicting scan frequency in elk.**
(DOCX)
