# Supplementary material for: Effects of Humans on Behaviour of Wildlife Exceed Those of Natural Predators in a Landscape of Fear
Source: PLoS One. 2012 Nov 28;7(11):e50611. doi: 10.1371/journal.pone.0050611 (PMC3509092; doi:10.1371/journal.pone.0050611)
Supplement: Table S1 — Sets of models predicting group vigilance and scan frequency in elk (ranked using DIC). (DOCX) [file pone.0050611.s001.docx]

**Table S1.** **Sets of models predicting group vigilance and scan frequency in elk (ranked using DIC).**

| *AIC rank* | *DIC rank* | *Dep. variable: arcsine square root [group vigilance], n = 424 elk groups* | *DIC* |
| --- | --- | --- | --- |
| **1** | **1** | **ln[herd size] + land-use/season + dist. nearest tree cover**  **+ dist. nearest road (>12 vehicles per day)** | **29.9** |
| 4 | 2 | ln[herd size] + land-use/season+ dist. nearest road (>12 vehicles per day)  + wolf RSF + grizzly bear RSF | 32.7 |
| 3 | 3 | ln[herd size] + land-use/season+ dist. nearest road (>12 vehicles per day)  + Terrain ruggedness | 44.0 |
| 5 | 4 | ln[herd size] + land-use/season + dist. nearest tree cover | 49.8 |
| 7 | 5 | ln[herd size] + land-use/season+ wolf RSF + grizzly bear RSF | 55.1 |
| 2 | 6 | ln[herd size] + land-use/season + dist. nearest road (>12 vehicles per day) | 55.3 |
| 8 | 7 | ln[herd size] + land-use/season+ Terrain ruggedness | 69.5 |
| 6 | 8 | ln[herd size] + land-use/season | 80.0 |
| 13 | 9 | ln[herd size] + wolf RSF + grizzly bear RSF | 159.1 |
| 10 | 10 | ln[herd size] + dist. nearest tree cover | 161.6 |
| 9 | 11 | ln[herd size] + dist. nearest road (>12 vehicles per day) | 161.8 |
| 12 | 12 | ln[herd size] + Terrain ruggedness | 170.7 |
| 11 | 13 | ln[herd size] | 181.8 |
| 14 | 14 | Intercept only | 367.0 |

| *AIC rank* | *DIC rank* | *Dep. variable: ln [scan frequency + 1], n = 870 focal elk* | *DIC* |
| --- | --- | --- | --- |
| **1** | **1** | **ln[herd size] + land-use/season +**  **dist. nearest road (>12 vehicles per day)** | **-11.6** |
| 3 | 2 | ln[herd size] + land-use/season + wolf RSF + grizzly bear RSF | -0.6 |
| 2 | 3 | ln[herd size] + land-use/season + inter-individual distance | 3.8 |
| 6 | 4 | ln[herd size] + land-use/season + age/sex class | 3.9 |
| 8 | 5 | ln[herd size] + land-use/season + Terrain Ruggedness | 7.5 |
| 4 | 6 | ln[herd size] + land-use/season+ dist. nearest tree cover | 11.6 |
| 7 | 7 | ln[herd size] + land-use/season + within-group position | 12.7 |
| 5 | 8 | ln[herd size] + land-use/season | 19.3 |
| 10 | 9 | ln[herd size] + wolf RSF + grizzly bear RSF | 140.4 |
| 9 | 10 | ln[herd size] + dist. nearest road (> 12 vehicles per day) | 142.4 |
| 11 | 11 | ln[herd size] + age/sex class | 152.5 |
| 15 | 12 | ln[herd size] + Terrain Ruggedness | 162.2 |
| 12 | 13 | ln[herd size] + inter-individual distance | 162.3 |
| 13 | 14 | ln[herd size] + dist nearest tree cover | 165.3 |
| 16 | 15 | ln[herd size] + within-group position | 168.2 |
| 14 | 16 | ln[herd size] | 174.0 |
| 17 | 17 | Intercept only | 302.2 |

Two sets of linear mixed effect models fit to predict group vigilance (upper panel) and scan frequency (lower panel) in elk observed in SW Alberta, Canada. Models were ranked using the Deviance Information Criterion *DIC*. Best predicting models (first rows, in bold) were the same regardless the use of DIC or AIC in model ranking.
