# Supplementary material for: Effects of Humans on Behaviour of Wildlife Exceed Those of Natural Predators in a Landscape of Fear
Source: PLoS One. 2012 Nov 28;7(11):e50611. doi: 10.1371/journal.pone.0050611 (PMC3509092; doi:10.1371/journal.pone.0050611)
Supplement: Table S2 — Selection of the best road variable predicting group vigilance in elk. (DOCX) [file pone.0050611.s002.docx]

**Table S2. Selection of the best road variable predicting group vigilance in elk**

|  | *AIC* | *rank* |  | *DIC* | *rank* |
| --- | --- | --- | --- | --- | --- |
| **Distance from the nearest road (> 12 vehicles per day)** | **118.6** | **1** |  | **29.9** | **1** |
| Distance from the nearest road (> 6 vehicles per day) | 128.8 | 2 |  | 41.3 | 4 |
| Distance from the nearest road (> 3 vehicles per day) | 129.6 | 3 |  | 42.1 | 6 |
| Density of roads (> 6 vehicles per day) within a 3 km buffer | 130.1 | 4 |  | 42.4 | 7 |
| Distance from the nearest road (> 48 vehicles per day) | 130.2 | 5 |  | 39.6 | 3 |
| Density of roads (> 3 vehicles per day) within a 3 km buffer | 130.4 | 6 |  | 42.6 | 8 |
| Density of roads (all roads) within a 3 km buffer | 130.4 | 7 |  | 42.6 | 9 |
| Density of roads (> 48 vehicles per day) within a 3 km buffer | 130.4 | 8 |  | 43.5 | 11 |
| Density of roads (> 12 vehicles per day) within a 3 km buffer | 130.9 | 9 |  | 43.3 | 10 |
| Density of roads (> 96 vehicles per day) within a 3 km buffer | 131.0 | 10 |  | 44.7 | 14 |
| Density of roads (> 24 vehicles per day) within a 3 km buffer | 131.1 | 11 |  | 43.7 | 13 |
| Distance from the nearest road (all roads) | 131.9 | 12 |  | 37.0 | 2 |
| Distance from the nearest road (> 24 vehicles per day) | 132.7 | 13 |  | 43.5 | 12 |
| Distance from the nearest road (> 96 vehicles per day) | 133.3 | 14 |  | 41.6 | 5 |

Linear mixed models predicting group vigilance in 424 elk groups observed in SW Alberta, Canada. Each model corresponds to the top-ranked one from each set of *a priori* models built to predict group vigilance starting from the same set of predictor variables and including only 1 of the 14 road variables at time (see Methods for details). Each top-ranked from each set of *a priori* models included as best predictors ln[herd size], Land-use/season, distance from the nearest tree cover, and one road variable. Models were named according to the road variable included. Among road variables, the distance from the closest road with a traffic volume of at least 12 vehicles per day was the best in predicting group vigilance, as confirmed by both the Akaike Information Criterion *AIC* and the Deviance Information Criterion *DIC*.
