# Supplementary material for: Effects of Humans on Behaviour of Wildlife Exceed Those of Natural Predators in a Landscape of Fear
Source: PLoS One. 2012 Nov 28;7(11):e50611. doi: 10.1371/journal.pone.0050611 (PMC3509092; doi:10.1371/journal.pone.0050611)
Supplement: Table S3 — Selection of the best road variable predicting scan frequency in elk. (DOCX) [file pone.0050611.s003.docx]

**Table S3.** **Selection of the best road variable predicting scan frequency in elk**

|  | *AIC* | *rank* |  | *DIC* | *rank* |
| --- | --- | --- | --- | --- | --- |
| **Distance from the nearest road (> 12 vehicles per day)** | **75.3** | **1** |  | **-11.6** | **1** |
| Distance from the nearest road (> 48 vehicles per day) | 82.3 | 2 |  | 4.1 | 2 |
| Density of roads (> 96 vehicles per day) within a 3 km buffer | 85.1 | 3 |  | 10.7 | 6 |
| Density of roads (> 24 vehicles per day) within a 3 km buffer | 85.9 | 4 |  | 10.3 | 5 |
| Distance from the nearest road (> 24 vehicles per day) | 86.2 | 5 |  | 9.4 | 3 |
| Density of roads (>48 vehicles per day) within a 3 km buffer | 87.3 | 6 |  | 12.4 | 7 |
| Density of roads (>12 vehicles per day) within a 3 km buffer | 88.6 | 7 |  | 13.1 | 8 |
| Density of roads (> 3 vehicles per day) within a 3 km buffer | 89.2 | 8 |  | 13.6 | 9 |
| Density of roads (all roads) within a 3 km buffer | 89.2 | 9 |  | 13.6 | 10 |
| Density of roads (> 6 vehicles per day) within a 3 km buffer | 89.4 | 10 |  | 13.8 | 11 |
| Distance from the nearest road (> 96 vehicles per day) | 89.8 | 11 |  | 10.1 | 4 |
| Distance from the nearest road (all roads) | 89.9 | 12 |  | 20.8 | 14 |
| Distance from the nearest road (> 6 vehicles per day) | 90.1 | 13 |  | 14.8 | 12 |
| Distance from the nearest road (at least 3 vehicles per day) | 90.1 | 14 |  | 14.8 | 13 |

Linear mixed models predicting scan frequency in 870 elk observed in SW Alberta, Canada. Each model corresponds to the top-ranked one from each set of *a priori* models built to predict scan frequency starting from the same set of predictor variables and including only 1 of the 14 road variables at a time (see Methods for details). Each top-ranked model from each set of *a priori* models included as best predictors ln[herd size], land-use/season, and one road variable. Models were named according to the road variable included. Among road variables, the distance from the closest road with a traffic volume of at least 12 vehicles per day was the best in predicting scan frequency, as confirmed by both the Akaike Information Criterion *AIC* and the Deviance Information Criterion *DIC*.
